# Combined Separator Based on a Porous Ion-Exchange Membrane for Zinc–Halide Batteries

**DOI:** 10.3390/membranes13010067

**Published:** 2023-01-05

**Authors:** Alexey Y. Rychagov, Yury M. Volfkovich, Valentin E. Sosenkin, Alexsandr F. Seliverstov, Marianna Y. Izmailova

**Affiliations:** A.N. Frumkin Institute of Physical Chemistry and Electrochemistry, Russian Academy of Sciences, Leninsky pr. 31, 119071 Moscow, Russia

**Keywords:** combined membrane, bromine crossover, self-discharge, zinc-bromine battery, asymmetrical conduction

## Abstract

In this work, we report on a comparative analysis of the bromine permeability for three separator groups under the operating conditions of a non-flow zinc–bromine battery. A new method for the synthesis of porous heterogeneous membranes based on a cation-exchange resin followed by treatment with tetrabutylammonium bromide is proposed. It was shown that the modified membrane significantly reduced the bromine permeability (crossover) with an acceptable increase in the ionic conductivity of the separator group. Leakage currents not exceeding 10–20 µA/cm^2^ were achieved, and the Coulomb efficiency was over 90%. The ionic conductivity (at AC) of a membrane soaked with water was compared for different pretreatment conditions. The frequency dependence of the membrane resistance is shown. The features of the conduction mechanism of the modified membrane are discussed.

## 1. Introduction

Polymer-based ion exchangers (ion exchange resins) were first synthesized in the 1930s [1]. Almost immediately after that, the technology of new ion-exchange materials was divided into dispersed and film directions. The most important direction is the use of membranes in electrophysical and electrochemical processes. Thanks to the creation of film membranes based on perfluorinated sulfonic acids [2,3], the industrial production of highly efficient fuel cells and portable electrolyzers has become possible. Membranes are used for electrochemical synthesis, in systems for the desalination and concentration of aqueous solutions, in electroanalytical methods, and in other areas of electromembrane technologies [4,5,6]. At this stage, developments in the field of electrochemical energy sources also widely use membranes of various types [7,8].

One of the most important tasks of green energy is the development of new eco-friendly and high-operational-safety electrical energy storage devices [9,10]. Due to these properties, zinc–halide batteries in aqueous electrolytes of zinc salts have attracted interest. Among the bromine-based redox battery technologies, zinc–bromine batteries have been one of the most studied and commercially scaled-up, both in non-flow cells and in flow configurations. Flow batteries have been manufactured by Primus Power and EnSync (Formerly ZBB) in the USA, RedFlow Ltd. in Australia, and ZBEST Power and Smart Energy in China [11,12]. For mobile applications that do not require external power sources and maintenance, non-flow batteries are more promising, such as those manufactured by Gelion Technologies in Australia and EOS Energy Enterprises in USA.

A zinc–bromine battery is a typical hybrid battery [13], because only the catholyte is a liquid and the anode is plated zinc.

Negative electrode: Zn^2+^ + 2e^−^ ⇌ Zn (1)

Positive electrode: 2Br^−^ − 2e^−^ ⇌ Br_2_ (2)

Summary reaction: ZnBr_2_ ⇌ Zn + Br_2_ (3)

During charging (forward arrows in Equations (1) and (2)), bromide ions are oxidized to bromine at the positive electrode, and at the same time metallic zinc is deposited as a thick film on the negative electrode. During discharging (backwards arrows in Equations (1) and (2)), the bromine is reduced to bromide ions while the deposited zinc is oxidized and dissolved into the electrolyte. Bromine possesses particular advantages such as a high reversibility of the bromine–bromide transition and the liquid state of aggregation. The bromine-to-bromide conversion potential allows the use of carbon electrode materials. The liquid state of aggregation allows the consumption of a large amount of bromine in small volumes at low pressure. The greatest toxic hazard when using such batteries in enclosed spaces and transport is molecular bromine, which has a low boiling point (58 °C). However, dissolved bromine molecules in zinc bromide solution form the complexes BrM_3_, BrM_5_ and BrM_7_; as a result, the concentration of Br_2_ in the electrolyte decreases, which significantly reduces the vapor pressure of bromine. Symmetrical and unsymmetrical tetraalkylammonium bromides are commonly used to reversibly bind bromine to form complexes. As a result of a complex formation, the concentration of molecular bromine in the electrolyte of a charged battery decreases to 0.1 M, which is lower than the solubility of bromine in the electrolyte. Correspondingly, the bromine vapor pressure over the electrolyte also decreases. Zinc is the most electronegative metal capable of being deposited from aqueous electrolytes, and at the same time, it is a low-toxicity material.

The most important problem that stands in the way of creating an efficient zinc–bromine battery is self-discharge, which is primarily due to the transfer of bromine through the separator. This problem is relevant for both flow-based and non-flow-based systems. One of the methods for reducing bromine leakage is the use of nonporous cation exchange membranes [14,15] (primarily perfluorinated) as a separator. However, the use of such membranes negatively affects the efficiency and charging stability, since it leads to the appearance of a concentration potential jump. In addition, since the electrolyte involves an acidic reaction (pH from 1 to 3), the predominant proton exchange properties of the membranes lead to the accumulation of bromic acid in the space of the negative electrode, which contributes to the release of hydrogen onto the zinc. Besides the membranes that prevent the diffusion of bromine to the zinc electrode, the use of high-viscosity gel electrolytes can decrease self-discharge. A highly concentrated gel electrolyte, ZnBr_2_ (20 M), with the addition of LiCl was applied to non-flow zinc–bromine without a membrane [8]. This approach led to a significant increase in the internal resistance and was close, in its physical meaning, to a decrease in the porosity of traditional separators. Increasing the viscosity of the electrolyte is one of other methods of reducing self-discharge. In any case, the leveling of the electrolyte concentration in a charged battery is necessary for its stable operation, which can be ensured only by the porosity of the separator. The increase in the porosity of the separator leads to an increase in bromine leakage, and a reduction in the internal resistance of the battery. Thus, a separator based on cation exchange materials with optimal porosity is needed.

However, the use of cation exchangers instead of the dielectrics used in traditional separators can significantly reduce the porosity while maintaining an acceptable decrease in the ionic conductivity of the separator. Good results for a non-flow zinc–bromine battery (efficiency close to 90%) were obtained using a porous composite membrane [16].

One of the problems of charging zinc–bromine batteries is the growth of zinc dendrites, which, as a rule, leads to an internal short circuit. Measures to prevent this phenomenon are consecrated in the works [17,18,19]. Besides a cation exchange membrane, the separator group of such batteries must contain a highly porous separator located on the side of the zinc electrode. Such an additional layer acts as a space for the zinc deposited during charging as well as being an electrolyte reservoir and separating the membrane from the metallic zinc.

The purpose of this work was to show the possibility of reducing the leakage currents associated with the transfer of bromine when using a separator based on a porous cation exchange membrane. In addition, the technological possibilities of manufacturing membranes using a KU-2-8 (analogue S-100) powder cation exchange resin as the main material was shown. This technology makes it possible to produce membranes with a controlled and evenly distributed porosity. One of the objectives of the study was an attempt to demonstrate a decrease in membrane permeability as a result of impregnation with a tetraalkylammonium salt of bromine (tetrabutylamonium bromide).

## 2. Materials and Method of Approach

### 2.1. Separator Selection and Pretreatment

The separator used in the experiment was a double-layered system that comprised a 160 µm thick highly porous (60–80%) nonwoven PP cloth (FS 2226, Velidon, made in Weinheim, Germany) and a 240 µm thick microporous cation exchange resin-based membrane (KU-2-8, CAS60177-20-7, Made in Chelyabynsk, Russia). The cation exchange resin KU-2-8 (similar to S-100, Lewatit) was granular material with a microporous structure of a styrene–divinylbenzene copolymer with a sulfonate functional group (SO_3_^−^). In comparative experiment, the membrane was replaced with a 60% porosity separator (polyethylene with silica gel, Grace brand, made in the Chicago, IL, USA), which was 220 µm thick. Detailed differences between each of the separator groups are described in the experimental section.

A porous cation exchange membrane was made by mixing an ion exchange resin (10–30 µm) with ethyl alcohol to obtain a suspension. A 5% (in terms of dry components) aqueous suspension of polytetrafluoroethylene was added to the resulting mixture. The ratio of the cation exchange resin and ethyl alcohol in suspension was 1:7. The resulting four-component suspension was filtered. After filtration, the mixture of the cation exchange resin and polytetrafluoroethylene was pressed for several cycles with movement until a pasty mass was obtained. Then, it was rolled out on a roller to the required thickness and dried in air to obtain the porous polymer base of the membrane. For the manufacturing of the membrane, resin in the H form was used. The membrane obtained in this way received the label KU-F5-240.

Similar heterogeneous membranes (MK-40 manufactured in Russia, Ralex CM manufactured in the Czech Republic, etc.) are widely used in the technologies of deionization and concentration of aqueous solutions [20,21]. The main difference in the KU-F5-240 membrane from its commercial counterparts was the coarser grinding of the resin and the replacement of the binder, which allowed the creating of the necessary interparticle porosity.

### 2.2. Electrochemical Measurements

**Figure 1:**
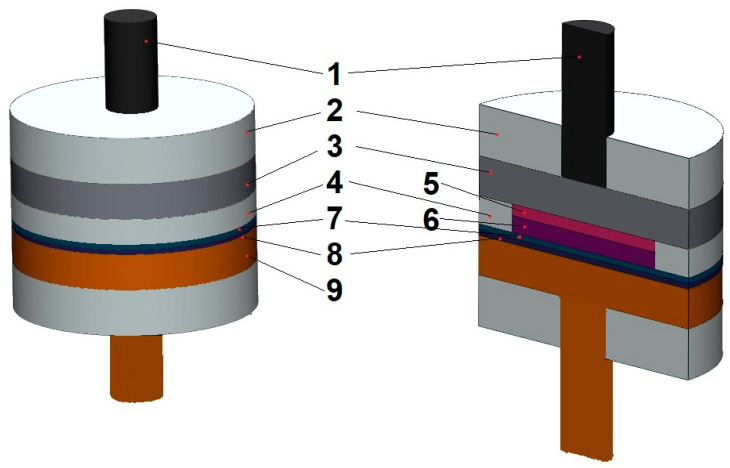
The scheme of two-electrode cell: 1—carbon current collector, 2—fluoroplastic ring, 3—graphite, 4—silicone ring, 5—graphite foil, 6—activated carbon fiber CH-900-20, 7—membrane, 8—separator, 9—Zn.

Electrochemical studies were carried out in a Teflon two-electrode cell simulating the operation of a non-flow zinc–bromine battery consisting of a zinc anode and a cathode based on activated carbon fiber CH-900-20 (Kuraray, Osaka, Japan) [22,23] separated by a separator group (Figure 1). The CH-900-20 electrode (weight 17 mg, area 2.1 cm^2^) was placed in a silicone ring closed with a graphite shim so that the bromine could only be transferred towards the zinc through the separator. The cell compression pressure was 1.5 kg/cm^2^. The separator group was located by the membrane towards the cathode. The electrolyte (3M ZnBr_2_) was in the pores of the activated carbon fiber and the separator group. Before assembling the cell, the activated carbon fiber and the separator group were soaked in the electrolyte for 3 days. Other features of the membrane preparation are indicated in the experimental section.

Charge–discharge, impedance, and voltammetric studies were carried out using a P-40X-FRA-24M potentiostat from Electro chemical instruments. The electrical conductivity was measured using a two-electrode circuit on a membrane compressed with a pressure of 1.5 kg/cm^2^ between two graphite shims. The modes of the electrochemical tests are indicated in the experimental section.

### 2.3. Standard Contact Porosimetry

The method of standard contact porosimetry (MSCP) was applied to the investigation of the porous structure membrane and separator [22]. This technique allowed us to establish the pore structure of the powder and compact materials in the widest possible range of pore radii (from 1 to 10^5^ nm). In [22,23,24,25], the MSCP technique is described in detail. The method of reference contact porosimetry was recognized by IUPAC as the correct method for studying porous structures [26]. Water was used as the measuring liquid in the work since the electrolyte of a zinc–bromine battery is an aqueous solution of ZnBr_2_.

## 3. Results and Discussion

### 3.1. Standard Contact Porosimetry

**Figure 2:**
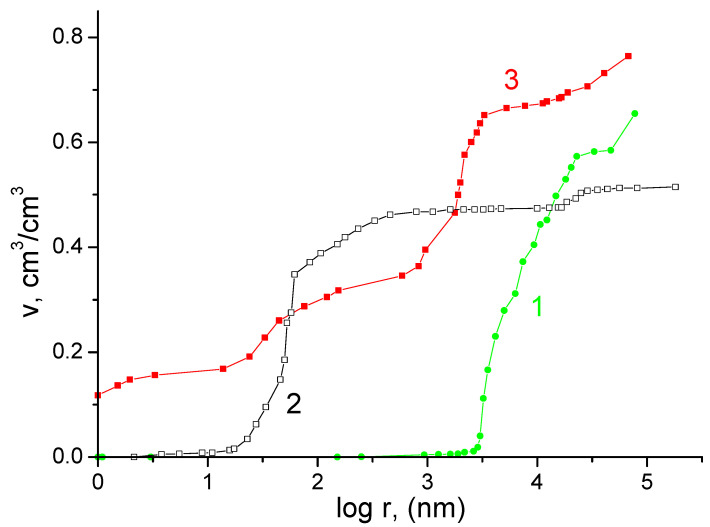
Integral porometric curves obtained by MSCP for: 1—separator FS 2226; 2—Grace separators; 3—KU-F5-240 membranes.

Figure 2 shows the integral porometric curves measured during the gradual drying of the samples flooded with water according to the MSCP. In pores with radii less than 0.5 µm (micro- and mesopores), the largest difference in the volumes of the existing pores was noted. So, for the Grace separator, this range of pore sizes contained more than 90% of the total volume; for the KU-F5-240 membrane, such pores made up less than half of the total pore volume; and for the FS 2226 separator, such pore sizes were completely absent.

**Figure 3:**
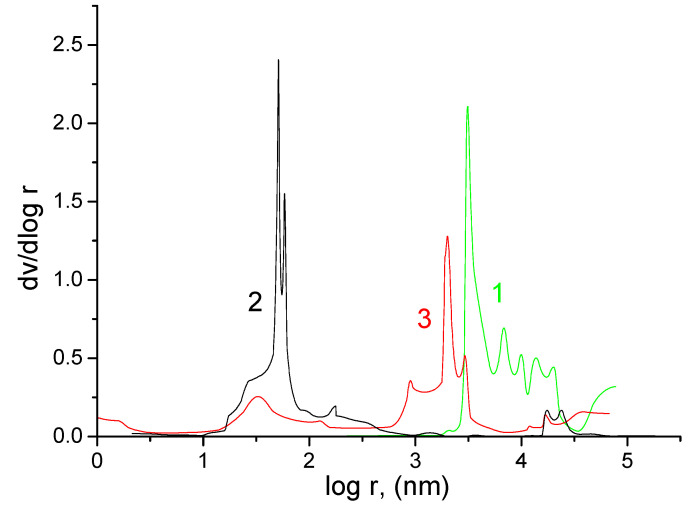
Differential curves of the distribution of pores along the radii obtained by MSCP for: 1—separator FS 2226; 2—Grace separators; 3—KU-F5-240 membranes.

Separator FS 2226 was highly porous, since the range of its pore radii was located in the region of macropore radii from ∼7 × 10^3^ to 10^5^ nm. The main maximum on the differential porometric curve for FS 2226 (Figure 3, curve 1) was in the region from ∼7 × 10^3^ to 7 × 10^4^ nm. The range of pore radii for the Grace separator was in the region from ∼10 to 10^5^ nm. On the differential porometric curve in Figure 3 for the Grace separator, two maxima are visible: the main maximum was from ∼10 to 700 nm, and a small maximum was noted from ∼1.6 × 10^4^ to 3.2 × 10^4^ nm. The presence of these two maxima was explained by the fact that this separator contained two components, namely polyethylene and silica gel. In the narrow region, from ∼70 to 90 nm, there is a very sharp peak (Figure 3, curve 2), which indicated the presence of a regular structure, apparently due to silica gel, in this region of pores.

The range of pore radii for the KU-F5-240 membrane (Figure 3, curve 3) was very wide, since it extended from nanopores, with r < 1 nm to r∼10^5^ nm. According to the differential pore size distributions (Figure 3), these curves were characterized by four main maxima: from ∼1 to 7 nm, from ∼10 to 100 nm, from ∼9 × 10^2^ to 8 × 10^3^ nm, and from ∼10^4^ to 10^5^ nm. The presence of several maxima in the region of large pores (a few microns in size) can be explained by the distributed dispersion of the particles of the cation exchange resin obtained during its grinding. The maximum in the mesopore region apparently determined the intrinsic pore size of the resin, while micropores were formed as a result of the swelling of the polymer structure [27,28,29,30].

From Table 1 it can be seen that the KU-F5-240 cation exchange membrane had the largest specific surface area (753 m^2^/g). Such a large value of the specific surface area measured by water was explained by the fact that ion exchange materials swell in water due to the hydration of ionogenic groups, which leads to the expansion of polymer chains [23]. As a result of this hydration, not only did the specific surface area increase but the porosity also increased. The porosity of the KU-F membrane (0.765 cm^3^/cm^3^) was greater than that of the FS 2226 and Grace separators.

### 3.2. Electrochemical Measurements

In order to conduct a comparative analysis of the main electrochemical characteristics of a zinc–bromine battery with a separator group based on a porous cation exchange membrane, three types of cells with different separators were assembled and studied. In cell1, a Grace separator was used as the main barrier for the transfer of bromine from the cathode to the anode space. Cell 2 was distinguished by the fact that the role of the above-mentioned separator was played by the porous membrane KU-F5-240, made on the basis of the cation exchange resin KU-2-8. In Cell3, the KU-F5-240 porous membrane was preliminarily kept in the electrolyte. The membrane in the protonated form was kept in a 0.01 M solution of tetrabutylammonium bromide (TBA) for two days, after which the surface excess of the solution was removed from it, and the membrane was transferred to the working electrolyte (3M ZnBr_2_) for three days.

**Figure 4:**
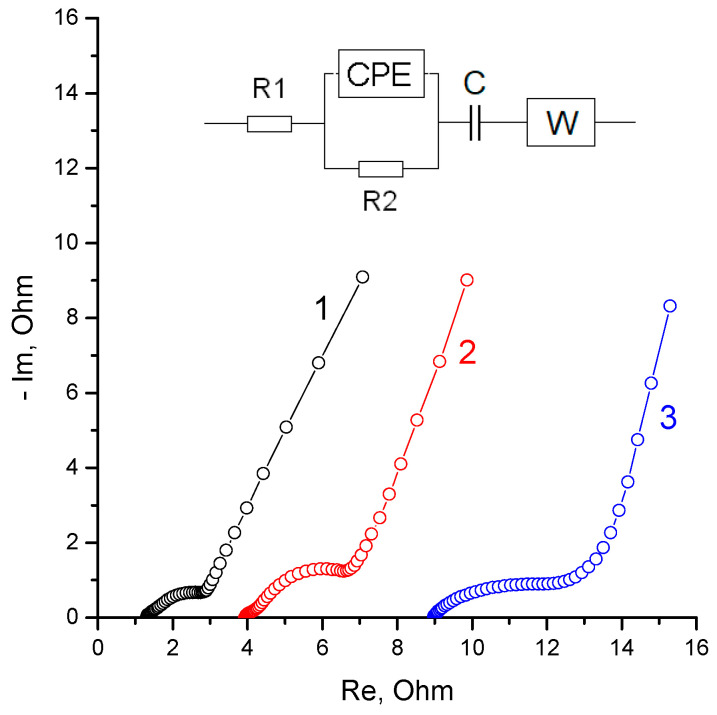
Nyquist diagram and equivalent electrical circuit for the initial state of cells 1, 2 and 3 measured in the frequency range from 100 kHz to 0.01 Hz.

After the assembly of each of the cells, primary impedance studies were carried out, which showed similar impedance spectra for all the cells (Figure 4). The main differences in the spectra were a systemic increase in the high-frequency resistance due to a decrease in the ionic conductivity of the separator group upon transition from the Grace mesoporous separator (cell 1) to the membrane KU-F5-240 (impregnated with TBA bromide (cell 3)).

The qualitative difference between the spectrum of cell 3 lay in the softer conversion of the medium frequency region (zinc electrode impedance) to the low frequency region (charging of the double electric layer on activated carbon). This may be due to a change in the mechanism of ion transport through the membrane impregnated with TBA bromide. Having swollen in a dilute solution, the cation exchange resin formed a significant volume of micropores capable of concentrating TBA bromide, which is almost insoluble in the electrolyte (3M ZnBr_2_). The observed rather high ionic resistance of the membrane (Figure 4, curve 3) was apparently due to the interaction of low-mobility TBA ions with the functional groups of the cation exchange resin.

**Figure 5:**
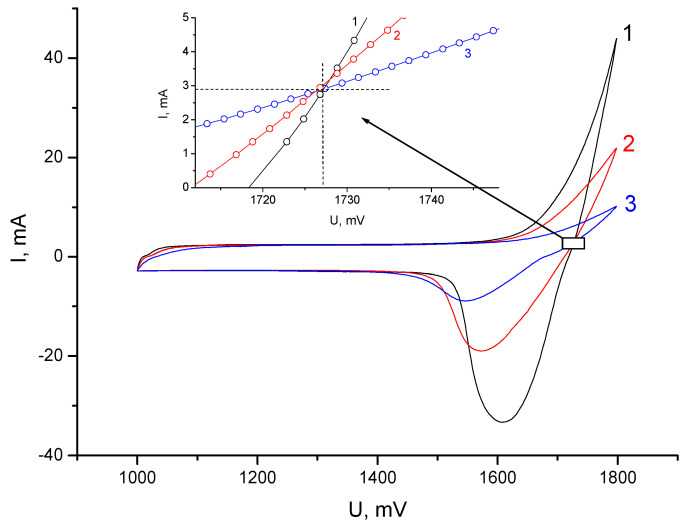
Cyclic voltammograms at a voltage sweep rate of 1 mV/s for: 1—cell 1; 2—cell 2; 3—cell 3. The inset shows an enlarged area of intersection of the curves.

The internal high-frequency resistance of the membrane had a noticeable effect on the shape of the cyclic voltammograms in the region of the main current-forming reaction, i.e., ZnBr_2_ ↔ Zn + Br_2_ (Figure 5). In this case, the charging currents of the electrical double layer remained unchanged for all the cells.

The consequence of the comparative analysis of the cyclic voltammograms was the determination of the point of optimal charge voltage (1727 mV), above which hydrogen evolution on the zinc electrode contributed to leakage currents (inset, Figure 5). In general, the release of hydrogen during the deposition of zinc on metallic zinc from aqueous solutions of ZnBr_2_ strongly depends on the concentration of the electrolyte and the presence of impurities in it. In this work, we assumed that the contribution of hydrogen released during charging (hydrogen leakage) to the total losses was insignificant and constant for all the compared experiments.

**Figure 6:**
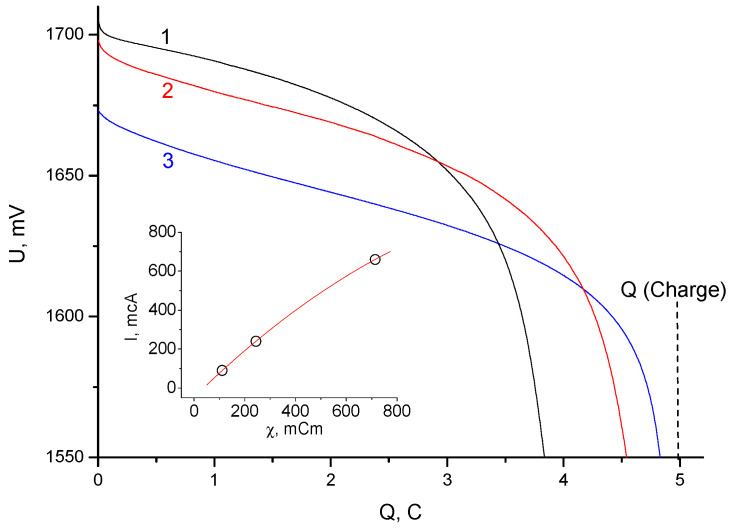
Discharge galvanostatic curves (with a current of 5 mA) obtained after charging the cells by 5 C for: 1—cell 1; 2—cell 2; 3—cell 3. The inset shows the dependence of the leakage current (self-discharge) on the electrical conductivity of the separator group at a frequency of 10 kHz.

After taking the voltammograms, the cells were subjected to charge–discharge cycling with a current of 5 mA per cell (the area of the working electrode was 2.1 cm^2^), while the charge was limited by the amount of electricity and the discharge limited by the voltage. Figure 6 shows a comparison of the stable discharge curves obtained after charging the cells by 5 C. The losses of the Coulomb capacitance, associated primarily with the transfer of bromine through the separator, were determined by the differences between charge (5 C) and discharge (Figure 6).

Based on the data in Figure 6, a calculated estimate of the average bromine leakage current through the separator was obtained for the three types of separator groups. To achieve this, the difference between the charge and discharge was divided by the time spent by the free bromine in the space of the positive electrode, that is, in the first approximation, by the total cycle time. The inset in Figure 6 shows the dependence of the bromine leakage current on the electrical conductivity of the separator group obtained from the impedance measurement data (Figure 4).

In this case, the electrical conductivity was defined as the reciprocal of the high-frequency (10 kHz) resistance (the real component of the impedance). As can be seen from the data obtained (inset, Figure 6), the dependence was close to linear, passing through the origin of coordinates (although there were also noticeable deviations towards the ascending parabola). This form of dependence is more typical for separators made from materials that do not have their own ionic conductivity. However, it should be taken into account that in this case the calculated electrical conductivity was determined not only by the resistance of the membrane layer but also included the entire cell impedance. If the curve shown in the tab of Figure 6 is corrected by subtracting the cell’s own impedance (not less than 0.5 Ohm), the shape of the curve will change significantly, approaching the root function. In addition, it should be taken into account that the data (inset, Figure 6) on leakage currents were obtained from short cycles (about 0.5 h) at small charging depths (less than 50%). The ionic conductivities of the individual elements of the separator group are given in Table 2. Here, it is necessary to point out that conductivities measured by a two-electrode scheme between smooth electrodes, as a rule, give somewhat underestimated values.

Under these conditions, there was a contribution from hydrogen leakage and the inertia associated with bromine remaining in the separator group from the previous cycle. When comparing data on electrical conductivity and leakage currents with porosimetry data, it can be concluded that the total porosity of the membrane did not determine the effective electrical conductivity and bromine permeability. This behavior may be due to the heterogeneous structure of the membrane and the specific adsorption properties of the cation exchange resin itself. In any case, the data obtained (Figure 6) showed that the maximum inhibition of Bromine transfer was observed for cell3.

**Figure 7:**
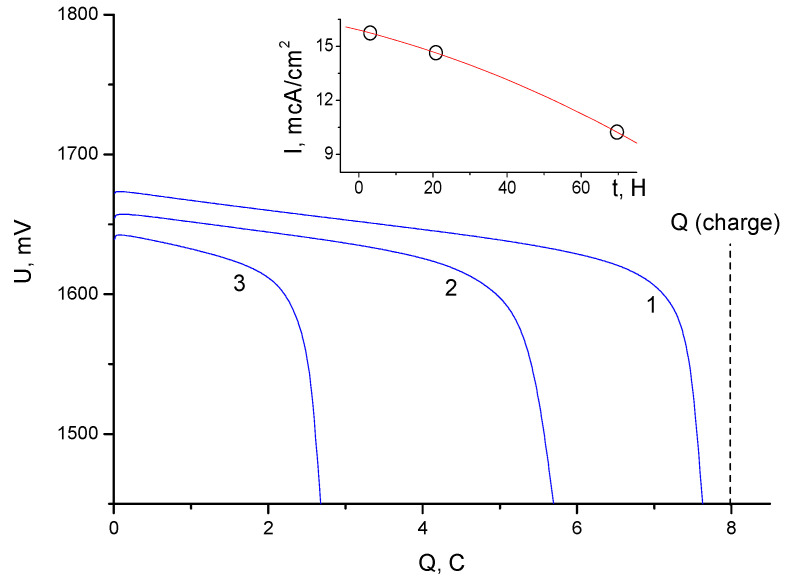
Discharge galvanostatic curves (with a current of 5 mA) obtained after charging cell 3 by 8 C for different exposure times after charging: 1—exposure for 2.5 h; 2—exposure for 21 h; 3—exposure for 69 h. The inset shows the dependence of the specific leakage current on the exposure time.

Since cell 3 showed the least leakage, an additional study was carried out for it at a deeper charge and with various shutter speeds of the charged cell (Figure 7). The exposure effect is shown on the tab in Figure 7. The average leakage currents per unit membrane surface ranged from 20 to 10 µA/cm^2^ and depended on the concentration of bromine in the space of the positive electrode.

**Figure 8:**
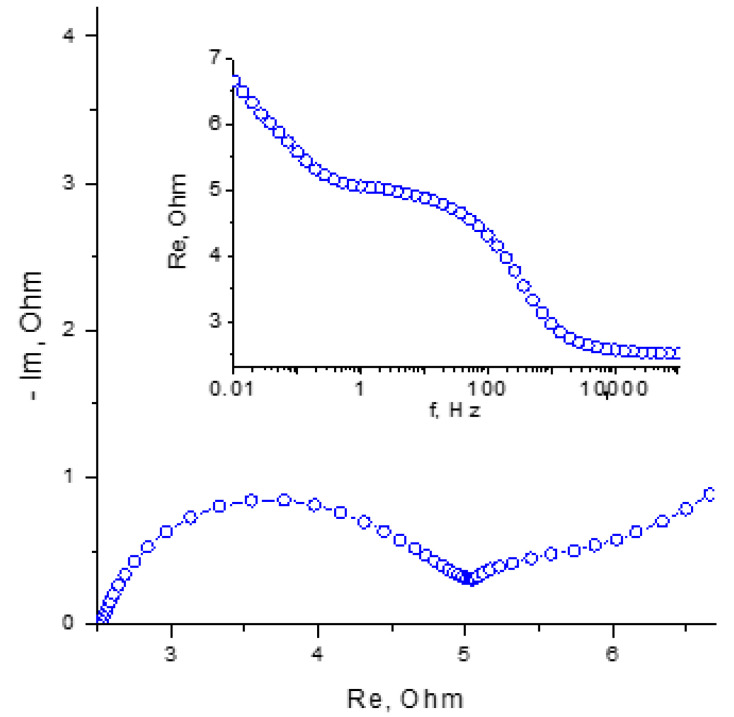
Nyquist diagram for charged cell 3 at 1720 mV in the frequency range from 10 kHz to 0.01 Hz. The inset shows the dependence of the real component of the impedance on the logarithm of the frequency (Bode diagram) in the range from 100 kHz to 0.01 Hz.

The modification of the membrane by impregnation with TBA bromide was carried out in order to bind the molecular bromine into low-mobility complexes, which should lead to a decrease in the membrane permeability. (Previously, this type of modification was proposed to give the MK-40 membrane an asymmetric diffusion permeability [31].) An important observation of the experiments performed was a gradual decrease in the measured resistance during cycling. This was especially pronounced under the conditions of deep charging. The spectrum of the impedance and the dependence of the real component of the impedance on the logarithm of the frequency after charging cell 3 by a charge of 8 C is shown in Figure 8.

Comparing the impedance spectra of cell 3 (Figure 4 and Figure 8), one can note a significant decrease in the internal resistance, which was a result of the cycling. The high-frequency resistance of the freshly assembled cell (Figure 4, curve 3) exceeded the resistance of the entire frequency interval for the charged cell after stabilization cycling (Figure 8). This effect may be a consequence of the gradual interaction of the molecular bromine with the poorly soluble TBA bromide in the electrolyte. As a result, insoluble complexes capable of transporting bromine ions could have formed in the pores of the membrane, increasing the overall electrical conductivity of the membrane.

**Figure 9:**
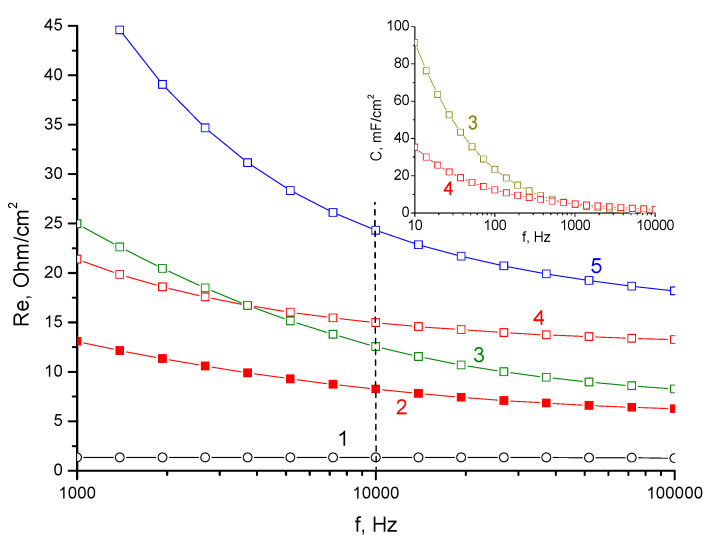
Dependence of resistance on the logarithm of frequency for: 1—intrinsic resistance of the measuring cell; 2—membrane soaked in 3M ZnBr_2_; 3—membrane kept in water; 4—sample 2 washed in water; 5—membrane from battery cell 3 washed in water. The inset shows dependence of the measured capacitance for samples 3 and 4 on the logarithm of the frequency.

Indirectly, this was indicated by a decrease in the resistance of cell 3 in the charged state. One of the factors affecting the overall conductivity of the considered membrane in a ZnBr_2_ solution can also be proton transfer, which is determined by the acidic properties of the electrolyte (pH about 1.5). Despite the higher concentration of Zn^2+^, doubly charged zinc cations must lose competition in the exchange kinetics of highly mobile hydrogen ions. Therefore, for a more detailed understanding of the mechanisms that determine the electrical conductivity of the KU-F5-240 membrane, a direct measurement of the resistance (separately from the battery cell) was carried out for different conditions of membrane pretreatment (Figure 9).

The primary comparative analysis of Figure 9 showed the similarity of curves 2 and 4, that is, the membrane in the electrolyte and the same membrane after washing it in water. The shift in resistance by about 7 Ω/cm^2^ was most likely due to the ionic conductivity of the electrolyte in the pores of the membrane. A comparison of membranes flooded with water in the H form (curve 3, Figure 9) and Zn form (curve 4, Figure 9) showed that the conductivity of the proton form exceeded the zinc-substituted form only in the high-frequency region. The significant increase in the resistance of the H form with a decrease in the frequency may be due to the high energy of interaction of hydrogen ions with the surface of the graphite washers that compressed the membrane, which led to an increase in polarization (not ohmic) resistance. This was confirmed by the difference in the capacitive components obtained by measuring the impedance of samples 3 and 4 (inset, Figure 8). Sample 5 (membrane from battery cell 3 in water) showed the highest resistance, which was due to the substitution of part of the functional groups with low-mobility TBA cations. However, the qualitative similarity of curves 5 and 3 allowed us to make an assumption about the possible contribution of proton conductivity to the total conductivity of the membranes modified with TBA bromide

The total conductivity of the membrane, which showed the minimum leakage currents according to bromine (cell 3), consisted of several components. This was, first of all, the cationic surface conductivity of functional groups, primarily with respect to zinc ions and partially due to proton exchange. The second most important mechanism was the ionic conductivity of the electrolyte located in the pores of the membrane. As a result of membrane impregnation, the contribution of this mechanism could have been significantly reduced due to blocking by insoluble forms of TBA bromide and its complexes with molecular bromine. The third possible mechanism of charge transfer across the membrane was the transfer of bromine ions through the insoluble TBA bromide complexes, which, under these conditions, were a solid anionic conductor.

## 4. Conclusions

According to the authors, the optimal structure of the separator group of zinc–halide batteries should contain a large-pore separator located on the anode (zinc electrode) side and a porous membrane capable of conducting zinc cations and halogen anions from the cathode side. The contribution of ionic conductivity through the pores of the membrane should not exceed the intrinsic membrane conductivity.

As a result of the work carried out on the use of porous ion-conducting membranes as part of a combined zinc–bromine battery separator, the following results were obtained:

- It was shown that the cation exchange membrane made of a highly dispersed resin (KU-2-8, similar to S-100) with a binder of 5% polytetrafluoroethylene significantly slowed down the transfer of bromine to zinc (reduced the bromine leakage current) compared to a traditional mesoporous separator. Significantly lower bromine leakage currents were observed in the cells with a membrane (KU-F5-240), despite the higher porosity in the flooded state.
- It was shown that the electrical conductivity of the water-flooded membrane (KU-F5-240) in the H form strongly depended on the impedance measurement frequency and exceeds the membrane in the Zn form only for the high-frequency region.
- A method for modifying a porous cation exchange membrane (KU-F5-240) by impregnation in a dilute solution of TBA bromide was proposed and tested, due to which the leakage current for bromine was further reduced to values of 10–20 μA/cm^2^. Such leakage currents made it possible to achieve a high Coulomb efficiency (more than 90%) under the conditions of sufficiently long cycles. An analysis of the electrochemical behavior of the cells suggested that the modification of the porous cation exchange membrane led to the formation of a combined (matrix) cation–anion exchange membrane. The impregnation technique, in this case, made it possible to regulate the most important ion exchange characteristics of the membrane. Despite the fact that the mechanism of charge transfer on the combined membrane was not studied in this work, the asymmetric nature of the charge and discharge modes was noted.
- With a relatively high internal resistance of the battery cell, the maximum discharge current significantly exceeded the charge current.
- Based on the results of the work, a conclusion was made about the prospects of using modified ion exchange membranes in zinc–halide batteries.

## Figures and Tables

**Table 1:** Main characteristics of porometric measurements for FS 2226 separator, KU-F5-240 membrane, and Grace separator.

Sample	Porosity, cm^3^/cm^3^	Specific Surface Area of Mesopores, m^2^/g	Total Specific Surface Area, m^2^/g	Average Pore Radius, nm
FS 2226	0.655	0	0.8	13,550
KU-F5-240	0.765	158	753	5031
Grace	0.515	40.2	40.5	2320

**Table 2:** Ionic conductivity of individual elements of the separator group measured by a two-electrode circuit (between graphite shims) at a pressure of 1.5 kg/cm^2^.

No	Type	Ionic Conductivity, mS/cm
1	Grace separator, in electrolyte	3.40
2	FS 2226 separator, in electrolyte	3.15
3	KU-F5-240 membrane, in electrolyte	3.55
4	Modified TBA membrane, in electrolyte	2.84
5	KU-F5-240 membrane, in water (N-form)	1.82
6	Modified TBA membrane, in water (N-form)	1.08

## Data Availability

Not applicable.
